# Accuracy of ultrasonographic transcerebellar diameter for dating in third trimester of pregnancy in Nigerian women: a cross-sectional study

**DOI:** 10.1186/s12880-025-01634-z

**Published:** 2025-03-25

**Authors:** Okechukwu Uche Ofoegbu, Nicholas Irurhe, Tersur Terry Saalu, Oluwaseun Emmanuel Familusi, Charity Opeoluwapo Maduagu, Lucky Enajite Tietie, Olaniyi Araotan Kusamotu, Ochuwa Adiketu Babah

**Affiliations:** 1 Department of Obstetrics and Gynaecology, Lagos University Teaching Hospital, Idi-Araba, Lagos, Nigeria; 2 Faculty of Clinical Sciences, College of Medicine, University of Lagos, Idi-Araba, Lagos, Nigeria; 3 Department of Radiodiagnosis, Lagos University Teaching Hospital, Idi-Araba, Lagos, Nigeria; 4 Department of Global Public Health, Karolinska Institutet, Stockholm, Sweden

**Keywords:** Ultrasound scan, Obstetrics, Transcerebellar diameter, Femur length, Head circumference, Abdominal circumference, Biparietal diameter, Dating, Gestational age, Accuracy

## Abstract

**Background:**

Accurate prediction of foetal gestational age is of critical importance as it can positively affect the outcome of pregnancy. Routine sonographic estimation of gestational age using biparietal diameter, head circumference, abdominal circumference and femur length is popular but has limitations especially when used as a singly or in late pregnancy. Often pregnant women in low-middle-income countries like Nigeria register for antenatal care late in pregnancy, necessitating the need for a single, cost-effective parameter that requires minimal skills to measure gestational age accurately in late pregnancies. This study examined the accuracy of ultrasonographic transcerebellar diameter compared to other foetal biometric parameters for dating in third trimester of pregnancy.

**Methodology:**

An analytic cross-sectional study conducted at Lagos University Teaching Hospital, Idi-Araba, Lagos, on 110 pregnant women in their third trimester. Data was collected using an interviewer administered questionnaire. Transabdominal ultrasound scan was done to determine the gestational age by measuring the biparietal diameter, head circumference, abdominal circumference, femur length and transcerebellar diameter. Spearman’s correlation coefficient was used to determine the correlation between the biometric measurements; Accuracy was determine using gestational age from menstrual date as gold standard and comparisons made using Chi square test.

**Results:**

Mean age of participants was 31.5 ± 5.8 years; mean gestational age 236 ± 25 days. Compared to biparietal diameter, head circumference, abdominal circumference, and femur length, transcerebellar diameter correlates best with gestational age (*r* = 0.8837, *p* < 0.001). At an error margin of ± 2weeks, transcerebellar diameter had a high predictive accuracy of 84.6%, though significantly less than that for abdominal circumference alone, 86.4% (*p* = 0.003), and also less than that for all four well known foetal biometric parameters (biparietal diameter, head circumference, abdominal circumference, and femur length) combined, 85.5% (*p* < 0.001).

**Conclusion:**

Transcerebellar diameter has a better correlation with gestational age than other routine foetal biometric parameters and has high predictive accuracy for dating in third trimester of pregnancy. It may thus play a relevant role in low resource settings where there is shortage of staff and limited skills in obstetric ultrasonography.

**Clinical trial number:**

Not applicable for this study.

## Introduction

The precise knowledge of the gestational age in modern clinical practice cannot be over emphasised as it is the mainstay of an Obstetrician’s ability to successfully manage a pregnant woman from conception to delivery. Having this knowledge and bearing in mind the different peculiarities associated with each pregnant woman aids in the timely application of appropriate interventions required to ensure a successful pregnancy outcome. High incidence of perinatal mortality has been noted in patients whose gestational age is not accurate or unknown [1]. This uncertain gestational age can result in iatrogenic prematurity and its sequelae [2]. Pregnancy dating is a fundamental component of antenatal care [3]. Precise assessment of gestational age is required to optimize provisions made for obstetric interventions and conveyance area for preterm births. It is likewise a prerequisite to recognize and appropriately manage foetal development abnormalities.

In addition, exact assessment of gestational age is needed to give sensible worldwide estimates of preterm birth and intrauterine growth restriction [4]. Furthermore, wrong dating is fraught with difficulties in diagnosing small for gestational age and foetal growth restriction, as well as challenges in decision-making regarding labour induction for pregnancy complications like gestational diabetes, pregnancy-induced hypertension, intrahepatic cholestasis of pregnancy, and post-term pregnancy. In developing countries like Nigeria where the prevalence of preterm birth and intrauterine growth restriction is high, determining gestational age precisely may involve an assessment of numerous components [5]. For example, last menstrual period is frequently obscure, and monthly cycles can fluctuate in length [6]. Symphysio-fundal height can be a deceptive measure of gestational age as a result of variety in maternal adiposity, intrauterine growth restriction, uterine fibroids, multiple pregnancies or malpresentation [7]. 

The first trimester ultrasound using crown rump length measurements is viewed as the gold-standard method for estimating gestational age [8], but ultrasound early in gestation might not be routinely available in low-middle-income countries because many pregnant women do not present early for antenatal care [3, 9–15]. The mean gestational age at initiation of antenatal care ranged from 20.3 to 23.6 weeks in a study carried out in the South-South region of Nigeria; commonest reason for late initiation of antenatal care being misconception on the right time to commence antenatal care [3, 9]. 

Currently the parameters which are being used in obstetric ultrasonography for evaluating gestational age in second and third trimesters of pregnancy include biparietal diameter (BPD), abdominal circumference (AC), head circumference (HC) and femur length (FL). These four parameters are often used in combination to improve the accuracy of measurements in most settings. As gestation progresses, these biometric measurements become less precise for assessing gestational age, especially when there is a pathological growth restriction [16, 17]. In low-middle-income countries, where 19·3% of infants are born small-for-gestational age, the assumption that foetal size predicts gestational age probably will not be substantial [18]. We hypothesize that transcerebellar diameter (TCD) may be a reliable marker for estimation of gestational age since the cerebellum is not liable to change in its form and size because it lies protected inside the posterior fossa surrounded by dense petrous and occipital bones [19]. 

The value of TCD for determining gestational age in late pregnancy is already established by some studies [20–22]. The purpose of this study is to establish a nomogram for the prediction of gestational age if TCD measurement in late pregnancy is known.

This study sought to determine the accuracy of ultrasonographic TCD measurements in estimating gestational age, compare the correlation between ultrasonographic TCD measurements and gestational age with that of other biometric measurements such as BPD, HC, AC, and FL and to develop a mathematical equation for estimating gestational age using TCD in the third trimester of pregnancy.

## Methodology

### Study setting

This study was conducted at the Departments of Obstetrics and Gynaecology and Radiodiagnosis of the Lagos University Teaching Hospital (LUTH), Surulere, Lagos, Nigeria.

### Study design

It was an analytic cross-sectional study conducted between 1st March 2022 to 31st August 2022.

### Study population

The participants comprised eligible and consenting pregnant women registered for antenatal care at the health facility. Included in this study were pregnant women who were sure of the date of their last menstrual period, had regular menstrual period prior to pregnancy, were within the gestational age 27–40 weeks, had singleton foetus, and did not use any oral contraceptive pills for at least two months before her last menstrual period. Excluded were pregnant women who were not in the third trimester of pregnancy, those with postdate pregnancy, those unsure of date of their last menstrual period, had a pregnancy complication, had multiple gestation, or had foetal malformation already detected on ultrasound scan.

### Sample size determination

Using Cochran’s formula and considering the proportion of foetuses with gestational age correctly predicted within 3 days of actual gestational age to be 93.3% in the third trimester of pregnancy using TCD measurement as found in a previous study [13], a sample size of 110 pregnant women was calculated to be adequate for this study at a confidence level of 95% and precision of 0.05 having adjusted for finite population correction and considering 10% non-response for missing data and other contingencies.

### Sampling method

A systematic sampling technique was used to select pregnant women in their third trimester that fit into the inclusion criteria. Based on an estimation that an average of 67 new pregnant women are registered for antenatal care at LUTH monthly as found in the antenatal clinic report, and assuming 50% of them to be eligible at enrolment or when they later enter the third trimester if registered earlier, and with data collection expected to run maximum of six months, the estimated total population from which sample was to be taken, N was estimated as *N* = 50% x (total number of newly registered pregnant women expected during the duration of data collection) which was 201. Required sample size for this study, n was calculated to be 110. Using the formula for sampling interval [23], k = N/n, where: k is the sampling interval, a sampling interval of two was estimated for this study. So, every second eligible patient in the clinic each day was selected for this research until the desired sample size was reached. Whether to start with the first eligible participant or with the second eligible participant, research code 001 was decided by doing simple balloting.

### Data collection

Eligible study participants had their data collected using a pre-tested semi-structured interviewer-administered questionnaire. Data collected included socio-demographic details such as age, marital status, occupation, level of education, obstetric and antenatal history, with foetal biometric measurements of HC, BPD, FL, AC, and TCD and their corresponding predictive gestational age obtained from the ultrasound machine.

Participants who meet the inclusion criteria were further counselled about the procedure and its potential benefits including possibility of contributing to scientific knowledge and improving obstetric care. Informed consent was obtained from each participant before inclusion in the study. Each participant was assigned a unique research identification number. This number was written on the case note of participants who had been enrolled to avoid duplicate data collection. All examination including the ultrasound scan was done with the woman well draped while ensuring minimal but optimum exposure and with a chaperone in attendance.

### Ultrasound technique and TCD measurement

GE Voluson P8 ultrasound machine (year of manufacture 2018) with curvilinear transducer was used for this study. A trans-abdominal scan was done using 3.5–5 MHz transducer frequency to ensure adequate penetration and resolution of the cerebellum measured. The measurements were taken using the electronic callipers of the ultrasound machine, making use of the freeze frame capacity. The Voluson P8 ultrasound machine uses Hadlock formula. The scan procedure was conducted by OUO under the supervision of the OAB (a Consultant Obstetrician) and NI (a Consultant Radiologist). OUO underwent a four-months re-training with the Consultant Radiologist as a refresher before commencing the research.

**Fig. 1:**
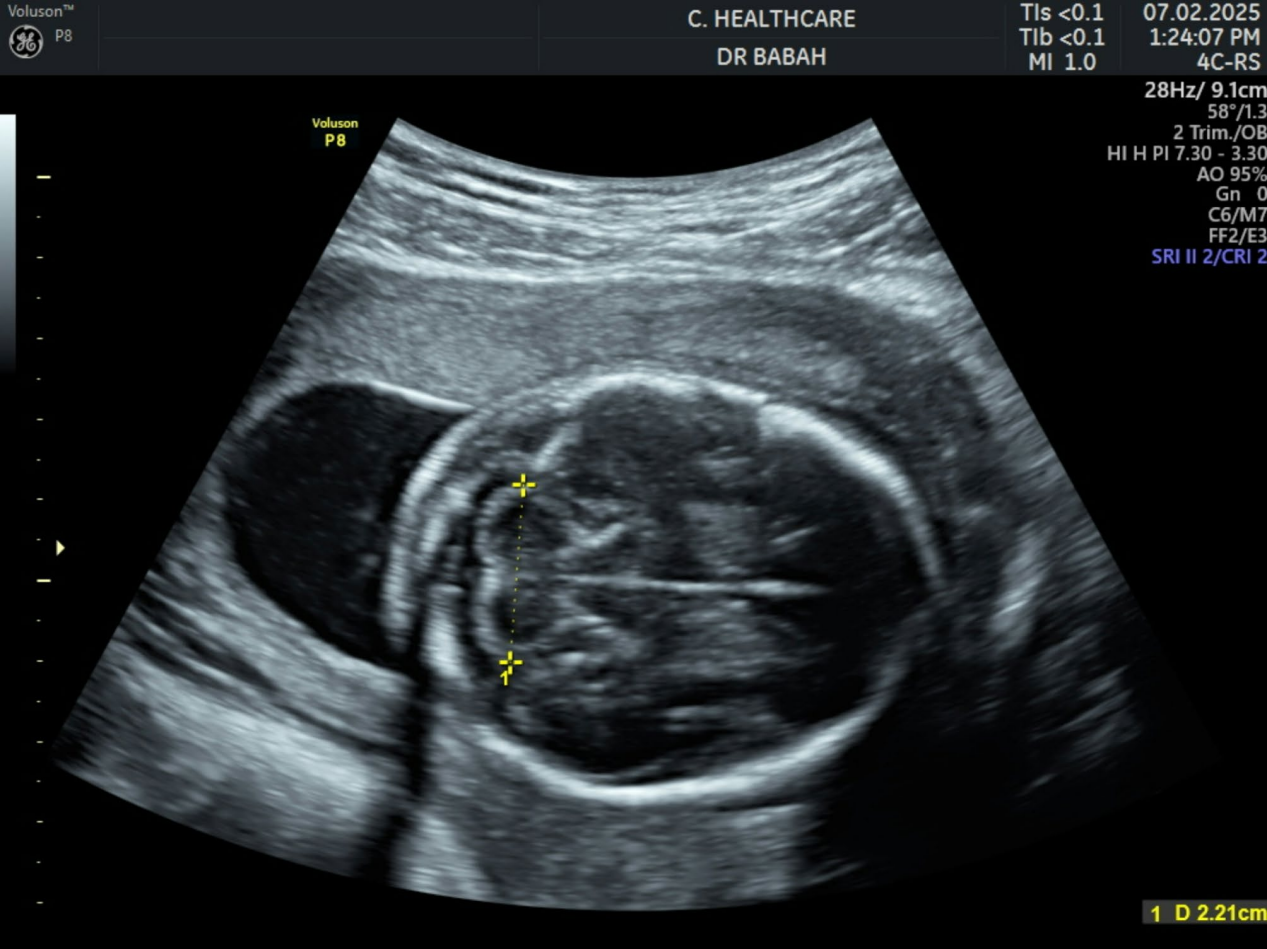
Landmark for measurement of transcerebellar diameter (TCD)

Procedure: In the scan room, each participant was again told about the purpose of the research, and she was allowed to ask questions. With the patient in supine position, the abdominopelvic region was exposed, and the ultrasound machine was set in an obstetric third trimester view. The foetal biometric measurements were taken as documented by Mishra et al. [24] using the freeze frame capacity technique. The foetal cerebellum was identified in the transverse view of posterior cranial fossa by using thalami, cavum septum pellucidum, and third ventricle as landmarks followed by rotational angulation of the transducer below the thalamic plane to view the cerebellar structure which appears like a butterfly. The TCD was measured by placing the callipers at the outer-to-outer margin measuring the widest diameter of the cerebellum where it appears characteristically as two lobules in either side of midline in the posterior cranial fossa. A single best and widest transverse diameter of cerebellum was recorded with this procedure [25]. Figure 1 below shows the landmark for TCD measurement. To improve precision of measurements, two independent measurements were taken by the lead (OUO) and second (OAB) authors, and the average of these values were used in data analysis to improve precision of measurements.

### Data analysis

Continuous variables such as age was presented as mean ± standard deviation, while discrete variables such as parity was presented as median and range, and categorical variables were presented as frequencies and percentages. Normality testing was done using Shapiro Wilks test. Correlations between foetal biometric measurements and foetal gestational age was computed using Spearman’s rank correlation coefficient. Accuracy was calculated as the sum total of all true positives and true negatives divided by the sum total of all true positives, true negatives, false positives, and false negatives and multiplied by 100. This was presented as percentage. Gestational age obtained from the date of the last menstrual period was used as the reference. Accuracy of the foetal biometric parameters were subsequently compared using Chi square test. Simple linear regression analysis was used to create a mathematical equation using the various foetal biometric parameters for predicting gestational age in third trimester pregnancy. Statistical significance was set at p-value less than 0.05. Statistical analyses were conducted using Stata version 16.1 (College Station, Texas USA). A two-tailed hypothesis was assumed at 95% level of confidence.

### Ethical consideration

Ethical approval was obtained from the Health Research Ethics Committee of the Lagos University Teaching Hospital (Approval No. ADM/DSCST/HREC/APP/4867) prior to commencement of the study. The study was conducted in accordance with the principles outlined in the Declaration of Helsinki. Informed consent was obtained from all participants prior to enrolment in the study.

## Results

The mean age of the participants was 31.5 ± 5.8years. They were enrolled at mean gestational age of 33.7 weeks ± 3.6 weeks. Their median parity was 1 (IQR: 0–2). Table 1 shows details of the sociodemographic characteristics of the pregnant women included in this study.

**Table 1:** Sociodemographic characteristics of the pregnant women included in this study

Sociodemographic characteristic	All participants, *n* = 110
Mean age ± S.D. (years)	31.5 ± 5.8
Mean gestational age at enrolment ± S.D. (weeks)	33.7 ± 3.6
Median parity ± IQR	1 (0–2)
	**Frequency (percentage)**, *n*** = 110**
**Marital status**	
Single	7 (6.4)
Married	103 (93.6)
**Ethnicity**	
Hausa	3 (2.7)
Igbo	31 (28.2)
Yoruba	69 (62.7)
Other	7 (6.4)
**Education**	
No formal education	2 (1.8)
Primary	2 (1.8)
Secondary	17 (15.5)
Tertiary	89 (80.9)
**Religion**	
Christianity	79 (71.8)
Islam	31 (28.2)
**Income**	
Less than NGN30,000	4 (3.6)
NGN30,000–100,000	48 (43.6)
NGN100,000–200,000	35 (31.8)
NGN200,000–300,000	12 (10.9)
Greater NGN300,000	3 (2.7)
No salary	8 (7.3)

The accuracy of estimating gestational age using TCD measurement was 58.2% to an error margin of ± 1week, 84.6% to an error margin of ± 2weeks, and 93.6% to an error margin of ± 3weeks. The accuracy of estimating foetal gestational age to an error margin of 1 week or 3 weeks with the use of TCD alone is comparable to the accuracy of using other estimating foetal biometric parameters (*p* < 0.05). TCD measurement for gestational age assessment at third trimester to an error margin of 2 weeks is significantly more accurate than BPD alone for dating pregnancy in second and third trimesters (accuracy of 84.6% versus 76.4%, *p* = 0.001). It is also significantly more accurate than using FL alone for dating in second and third trimesters (84.6% versus 80.9%, *p* < 0.001); but less accurate than AC alone (84.6% versus 86.4%, *p* = 0.003) and BPD, HC, AC, and FL combined (84.6% versus 85.5%, *p* = 0.001). Details on Table 2.

**Table 2:** Accuracy of transcerebellar diameter and other existing foetal biometric parameters for estimating gestational age in third trimester pregnancy

Ultrasound parameter	Error margin	Accuracy (%)	*p*-value
TCD	± 1 week	58.2	-
BPD		56.4	0.081
HC		50.9	0.259
AC		60.0	0.637
FL		58.2	0.090
BPD, HC, AC, FL combined		61.9	0.108
TCD	± 2 week	84.6	-
BPD		76.4	0.001*
HC		77.3	0.062
AC		86.4	0.003*
FL		80.9	< 0.001*
BPD, HC, AC, FL combined		85.5	0.001*
TCD	± 3 week	93.9	-
BPD		90.0	0.576
HC		90.9	0.507
AC		94.6	0.214
FL		90.0	0.507
BPD, HC, AC, FL combined		93.6	0.288

Accuracy is presented as percentage. *p*-value compares accuracy of each individual foetal biometric parameter – BPD, HC, AC, and FL alone with TCD. Comparison of accuracy was done using Chi square test. TCD – transcerebellar diameter, BPD – biparietal diameter, HC – head circumference, AC – abdominal circumference, FL – femur length

**Fig. 2:**
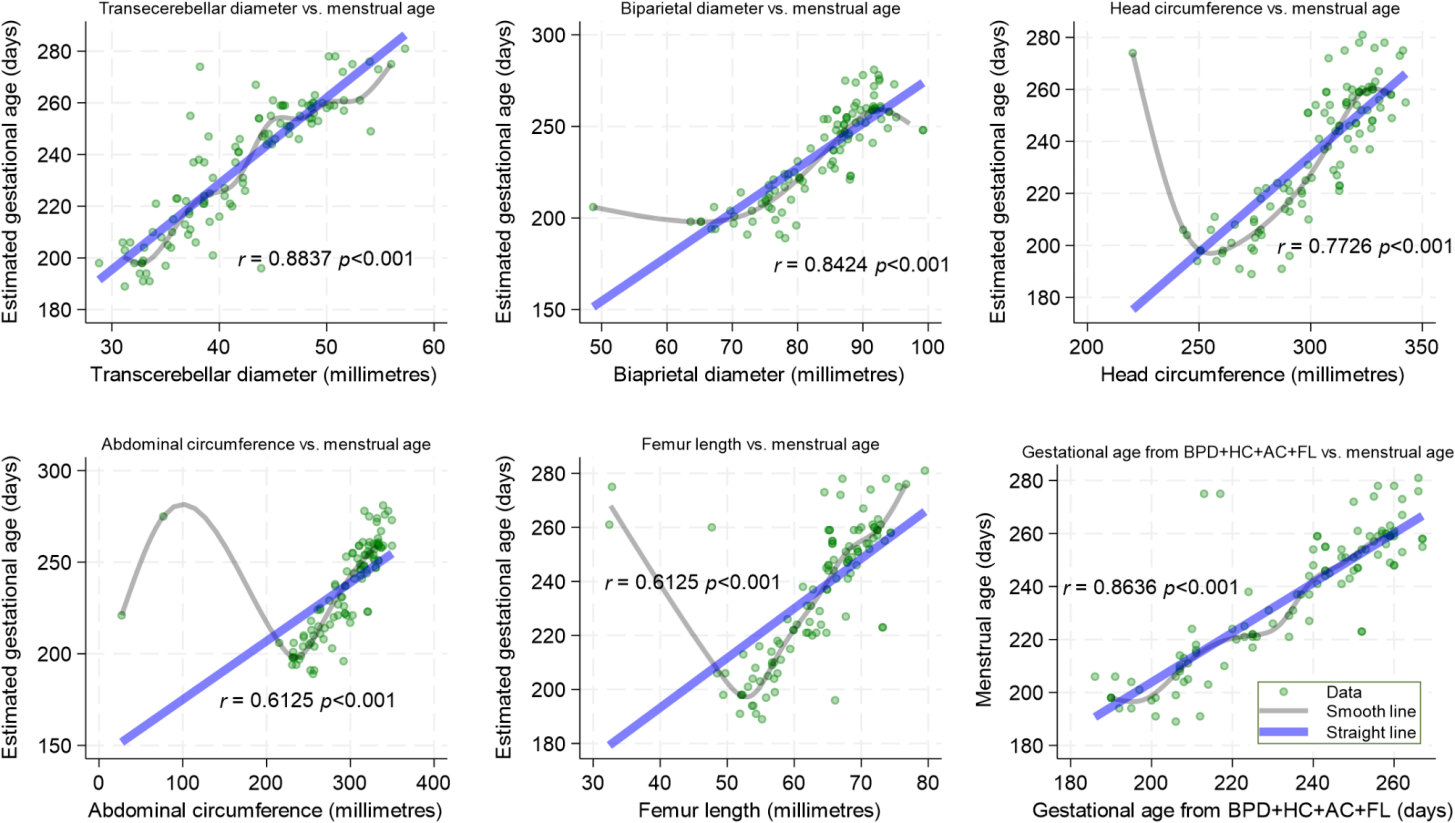
Correlation of transcerebellar diameter and other foetal biometric parameters with menstrual age in third trimester pregnancy

TCD measurements correlates best with foetal gestational age estimated from last menstrual period in the third trimester pregnancy compared to BPD, HC, AC, and FL. Figure 2 show scatter charts depicting the correlation between all biometric measurements and menstrual age (gestational age) of pregnancy. Looking at the smooth and straight line, there is evidence that for dating, the correlation between TCD and gestational age (menstrual age) is as good as that obtained from a combination of four biometric measurements BPD + HC + AC + FL.

Using simple linear regression modelling, a mathematical equation was derived for calculating gestational age when TCD measurement is known using the regression equation: y = a + bx; where: y is the dependent variable which here is the foetal gestational age in days, a is the intercept, b is the slope and x is the independent variable which here is the TCD measurement in millimetres. The linear equation obtained was:

GA = 95.7 + 3.3TCD

*Where: GA refers to gestational age.*

The accuracy of applying this formula for gestational age calculation was found to be 58.2% at an error margin of ± 1week, 84.6% at an error margin of ± 2weeks, and 93.6% at an error margin of ± 3weeks.

**Fig. 3:**
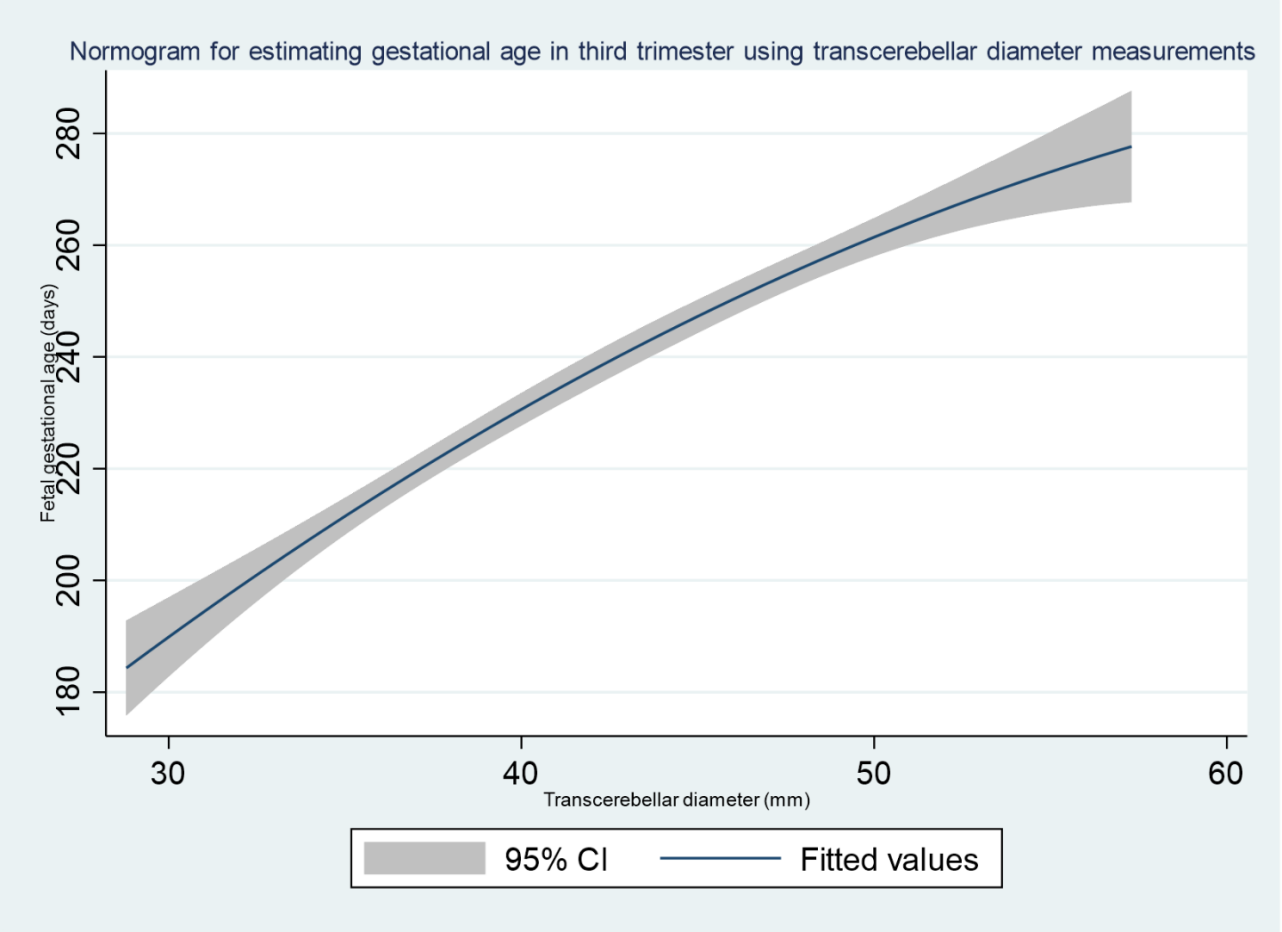
Nomogram for estimating gestational age in third trimester using transcerebellar diameter measurements

We developed a nomogram for gestational age estimation in third trimester pregnancy using TCD measurement, Fig. 3.

## Discussion

This study found TCD measurement alone to correlate best with gestational age calculated from last menstrual period compared to other foetal biometric parameters, BPD, HC, AC, and FL used singly or a combination of all four for dating in third trimester of pregnancy. It also has accuracies comparable to that of commonly used four parameter measurements (BPD + HC + AC + FL).

In this study, the accuracy of estimating gestational age at an error margin ± 2 weeks using TCD measurement was 84.6%; this was more accurate compared to BPD 76.4%, but less accurate compared to AC 86.4%. Ruqyyah et al. found a higher accuracy of 91.7% in third trimester of pregnancy, though the error margin was not stated [26]. Adeyekun AA et al. found a high predictive accuracy of 96.9% at error margin of *±* 12 days for gestational age determination using TCD [27]. This is higher than the accuracy of 84.6% at an error margin of ± 2 weeks which we obtained. Our findings are corroborated by several other studies which have consistency reported acceptable accuracies for use of TCD for dating at various error margins [28–31]. Like our study, Bekele et al. found TCD measurement to be more accurate than four parameter measurements [32]. 

The differences between studies regarding the degree of accuracy reported for the use of TCD for dating is partly because of the variation in inclusion gestational age of the women; Adeyekun AA et al. compared accuracy of using TCD for dating over a wider gestational age range which cuts across various trimesters [27]. In addition, variation in the skills of the sonologists might partly explain the differences in results obtained. Our study focused on dating in the third trimester of pregnancy because this is the period when issues relating to dating due to the inaccuracies of other existing methods become evident. Furthermore, it is in the third trimester of pregnancy that physicians and birth attendants often face dilemma related to wrong dating especially in women who present late for antenatal care or even in labour for the first time. Often, these women do not know the date of their last menstrual period and do not have an early scan report to confirm gestational age.

In addition, we found that TCD measurement had a strong positive correlation with menstrual age (gestational age) of pregnancy compared to BPD, HC, FL, or AC alone. Its correlation with menstrual age of pregnancy is comparable to the ultrasound derived gestational age applying Hadlock’s formula on the four well known biometric measurements, BPD + HC + AC + FL combined. The direction and magnitude of correlation in our study is consistent with the findings in earlier studies [27, 33–35]. We observed a linear association between TCD and menstrual age of pregnancy; this is supported by other studies [28, 36–39]. 

The mathematical equation derived in this study for use in our setting for estimation of gestational age when TCD has a predictive accuracy similar to that derived by Eze et al., and Reddy et al. in earlier studies [34, 35]. With the simple formula derived, gestational age can easily be calculated using TCD alone without going through the rigors of trying to do a complete ultrasound foetal biometric parameters scan in the advent of an emergency. Moreover, mastery of the skill in the measurement of TCD in third trimester can be achieved by trainee sonographers after a brief hands-on practice compared to the extensive training required to identify specific landmarks to facilitate accurate measurements of other popular foetal biometric parameters [40]. 

Unusual circumstances in which the accuracy of TCD measurements can be affected is in foetuses with structural abnormalities of the cerebellum such as Dandy walker malformation, a rare condition [41]. Other central nervous system disorders that may have mass effect on the foetal cerebellum like Blake’s pouch cyst, cerebellar hypoplasia, inferior vermian agenesis, myelomeningocele, and Joubert syndrome can affect TCD measurements [42, 43]. In addition, some chromosomal anomalies like trisomy 9, 13 and 18 can cause cerebellar hypoplasia resulting in an underestimation of gestational when TCD alone is used for dating [44, 45]. Pregnant women already diagnosed as having foetal anomaly were excluded from this study, and none of the women who participated in this study had an obvious foetal anomaly on ultrasonography. Furthermore, in advanced pregnancy near date or post-date, cranial bone calcification, reduction in amniotic fluid volume and foetal position may cause low penetration of the ultrasound beam, with reduced quality of the cerebellar image leading to inaccurate measurements especially when the foetal head is engaged [46]. 

Though recent advances in ultrasonography through application of artificial intelligence might help overcome some of these challenges, artificial intelligence-acquired foetal brain measurements (SonoCNS) has been found to have good reproducibility and repeatability for foetal skull measurements like BPD and HC, but performs poorly for intracranial measurements like TCD and cisterna magna diameter [47]. Superior to the SonoCNS for more accurate visualization of intracranial structures is the use of a semiautomated volumetric approach (5D CNS+™) which provides more reliable assessments even in presence of a cranial anomaly [48]. 

The major strength of our study is that the Voluson P8 BT16 model (year of manufacture 2018) ultrasound scanning machine which has an exceptionally good resolution for imaging was used for all the participants in this study. This helped to reduce instrument bias. In addition, the participants were selected using systematic sampling technique. This is a probabilistic sampling technique, and it helped to minimize selection bias. Establishing a nomogram and a formula from this study makes it possible to translate our research findings into practice especially in low resource settings with poorly developed health facilities. Often such facilities have skilled healthcare providers who have limited or no skills in ultrasonography. In addition, they may have lower versions of scan machines that cannot provide gestational age directly from TCD measurements. TCD measurements can be taken as a linear measurement in such situations, and the gestational age derived from the formula or nomogram we have presented here.

This study is limited by its use of last menstrual period to calculate the reference gestational age. The use of crown rump length has been found to be more accurate than menstrual age for dating pregnancy [49]. We decided to use menstrual age to date the pregnancies in this case because of some peculiarities in our environment; many of our women book late in pregnancy. The National Demographic Health Survey 2018 found that only 18% of pregnant women in Nigeria initiate antenatal care in the first trimester [50]. So it is relatively scarce getting women who initiated antenatal care at 7–10 weeks gestational age. To improve the accuracy of last menstrual period for estimating gestational age, we excluded from our sample pregnant women who were unsure of the date of their last menstrual period and those who had been on contraception in the last two months as some can cause menstrual irregularities leading to confusion in determining the actual date of their last period. We also excluded those who reported having irregular periods prior to conception because timing of ovulation is unpredictable in such instances. Some studies have also used gestational age obtained from last menstrual period as reference in similar studies [20, 21], while a few have used gestational age obtained from crown rump length in the first trimester which is the gold standard [22]. Either way, these studies have consistently reported high accuracy of TCD for dating in third trimester pregnancy. The accuracy of TCD for dating in the third trimester of pregnancy compared to other existing foetal biometric parameters raises hope that accurate dating can be feasible in the advanced pregnancy especially in low resource settings where skills in obstetric ultrasonography is limited.

## Conclusion

TCD has a better correlation with gestational age than other routine foetal biometric parameters and has high predictive accuracy for dating in third trimester of pregnancy. It may thus play a relevant role in low resource settings where there is shortage of staff and limited skills in obstetric ultrasonography. It will also be relevant in busy obstetric settings where a large pool of women may need to be scanned within a short time since TCD measurement alone requires less time to perform compared to the conventional measurement of four foetal biometric parameters (BPD, AC, HC, and FL) for dating.

## Data Availability

The dataset analysed for this research can be provided by OAB on reasonable request.

## Abbreviations

TCD: Transcerebellar diameter
BPD: Biparietal diameter
HC: Head circumference
AC: Abdominal circumference
FL: Femur length
